# Investigation of Phenolic Composition and Anticancer Properties of Ethanolic Extracts of Japanese Quince Leaves

**DOI:** 10.3390/foods10010018

**Published:** 2020-12-23

**Authors:** Vaidotas Zvikas, Ieva Urbanaviciute, Rasa Bernotiene, Deimante Kulakauskiene, Urte Morkunaite, Zbigniev Balion, Daiva Majiene, Mindaugas Liaudanskas, Pranas Viskelis, Aiste Jekabsone, Valdas Jakstas

**Affiliations:** 1 Institute of Pharmaceutical Technologies, Lithuanian University of Health Sciences, Sukilėlių av. 13, LT-50162 Kaunas, Lithuania; vaidotas.zvikas@lsmuni.lt (V.Z.); deimante.kulakauskiene@lsmu.lt (D.K.); urte.morkunaite@stud.lsmu.lt (U.M.); zbigniev.balion@lsmuni.lt (Z.B.); mindaugas.liaudanskas@lsmuni.lt (M.L.); pranas.viskelis@lsmu.lt (P.V.); aiste.jekabsone@lsmuni.lt (A.J.); 2 Laboratory of Biochemistry and Technology, Institute for Horticulture, Lithuanian Research Centre for Agriculture and Forestry, Kauno str. 30, LT-54333 Babtai, Lithuania; ievaurbanaviciute@yahoo.com; 3 Neuroscience Institute, Lithuanian University of Health Sciences, Eivenių str. 4, LT-50161 Kaunas, Lithuania; rasa.bernotiene@lsmuni.lt (R.B.); daiva.majiene@lsmuni.lt (D.M.); 4 Department of Pharmacognosy, Faculty of Pharmacy, Lithuanian University of Health Sciences, Sukilėlių av. 13, LT-50162 Kaunas, Lithuania; 5 Institute of Cardiology, Lithuanian University of Health Sciences, Sukilėlių av. 17, LT-50009 Kaunas, Lithuania

**Keywords:** *Chaenomeles japonica* leaves, phenolic compounds, glioblastoma, anticancer activity

## Abstract

Glioblastoma multiforme is an aggressive and invasive disease with no efficient therapy available, and there is a great need for finding alternative treatment strategies. This study aimed to investigate anticancer activity of the extracts of the Japanese quince (JQ) cultivars ‘Darius’, ‘Rondo’, and ‘Rasa’ leaf extracts on glioblastoma C6 and HROG36 cells. As identified by ultra high performance liquid chromatography electrospray ionization tandem mass spectrometry, the extracts contained three prevailing groups of phenols: hydroxycinnamic acid derivatives; flavan-3-ols; and flavonols. Sixteen phenols were detected; the predominant compound was chlorogenic acid. The sum of detected phenols varied significantly between the cultivars ranging from 9322 µg/g (‘Rondo’) to 17,048 µg/g DW (‘Darius’). Incubation with the extracts decreased the viability of glioblastoma HROG36 cells with an efficiency similar to temozolomide, a drug used for glioblastoma treatment. In the case of C6 glioblastoma cells, the extracts were even more efficient than temozolomide. Interestingly, primary cerebellar neuronal-glial cells were significantly less sensitive to the extracts compared to the cancer cell lines. The results showed that JQ leaf ethanol extracts are rich in phenolic compounds, can efficiently reduce glioblastoma cell viability while preserving non-cancerous cells, and are worth further investigations as potential anticancer drugs.

## 1. Introduction

Japanese quince (*Chaenomeles japonica* (Thunb.) Lindl. ex Spach), a representative of the *Rosaceae* Juss. family, has already been known in oriental folk medicine for about 3000 years [1]. This plant is a great source of secondary metabolites possessing various biological effects including anticancer activity [2,3]. For example, quince extracts have high amounts of triterpenes (such as ursolic and oleanolic acids) that are reported to decrease the viability of colon, breast, melanoma, lung, hepatic carcinoma, and other cancer cell types [4,5,6,7]. Furthermore, Japanese quince fruit extracts are rich in phenolic compounds, mostly flavonoids [8], that are also known for preventive and therapeutic anticancer potential [9]. Procyanidins and flavanols from Japanese quince fruits induce apoptosis and suppress invasiveness in human colon, prostate, and breast cancer cell cultures [10,11,12]. A recent study revealed that the extract of Japanese quince leaves reduces viability of colon cancer cells SW-480 and HT-29 to a greater extent compared to normal intestinal cells CCD-18 Co and CCD 841 CoN [13]. Such results encouraged us to investigate quince leaf extract efficiency on other cancer cell types.

Glioblastoma multiforme is one of the most aggressive and invasive cancerous diseases, and there are no efficient treatment options available [14]. The most common therapy is temozolomide, however the treatment is accompanied by severe side effects and the efficiency is poor [14]. Some promising results are achieved by applying plant-derived anticancer substances [15]. Therefore, it is important to continue investigating the new plant sources in order to find more efficient treatment or therapy complement for glioblastoma. Our previous research revealed that the leaves of three Japanese quince cultivars ‘Darius’, ‘Rondo’, and ‘Rasa’ are rich in phenols and triterpenes suggesting that the extracts might possess anticancer activity [16]. The current study aimed to perform a broader phenol analysis of the leaves of the same cultivars, and to investigate the effect of the extracts on the viability of glioblastoma HROG36 and C6 cells. In addition, to predict the potential level of cytotoxicity on healthy brain tissue, the effect of the extracts on viability of primary non-cancerous cultured brain cells was evaluated.

## 2. Materials and Methods

### 2.1. Chemicals

All the solvents, reagents, and standards used were of analytical grade. The following substances were used in the study: Ethanol 96% (*v*/*v*) (AB Strumbras, Kaunas, Lithuania), procyanidin C1, procyanidin B2, quercetin, hyperoside, avicularin, quercitrin, kaempherol 3-O-glucoside, luteolin 7-O-glucoside, phloridzin, formic acid, acetonitrile, (+)-catechin, (-)-epicatechin, rutin, isoquercitrin, chlorogenic acid, p-coumaric acid, caffeic acid, hydrochloric acid, Hoechst33342, propidium iodide, glucose, temozolomide, DMSO and KCl (Sigma-Aldrich, Steinheim, Germany), Dulbecco’s Modified Eagle Medium (DMEM) with Glutamax, foetal bovine serum, penicillin-streptomycin, Versene solution, antibiotic-antimycotic solution (Anti-Anti) were of Gibco brand and purchased from Thermo Fisher Scientific, Waltham, MA, USA. During the study, we used purified de-ionized water prepared with the Milli–Q^®^ (Millipore, Bedford, MA, USA) water purification system.

### 2.2. Plant Material and Extract Preparation

Japanese quince (*C. japonica*) leaves were collected in September 2018, after ripe fruits were harvested, from the garden of the Institute of Horticulture, Lithuanian Research Centre for Agriculture and Forestry, Babtai (55°60′ N, 23°48′ E). The leaves of each cultivar were collected from five shrubs, and frozen (at −40 °C) in a freezer with air circulation, and then lyophilized with a sublimator Zirbus 3 × 4 × 5 (ZIRBUS technology GmbH, Bad Grund, Germany), at a pressure of 0.01 mbar (temperature of condenser −85 °C) for 24 h. The lyophilized leaves were grounded to a fine powder with a knife mill GM (Retsch GmbH, Haan, Germany). Powdered leaf sample of each cultivar (2.5 g) was mixed with 50 mL of 40% ethanol, and extracted with ultrasonic bath Sonorex Digital 10 P (Bandelin Electronic GmbH & Co. KG, Berlin, Germany) for 40 min, at 60 °C, using 480 W ultrasonic power. The extracted samples were centrifuged and then filtered through filter paper (Watman no. 1). The Japanese quince ethanolic extracts (5 g/100 mL) were kept in a freezer (at −40 °C) in hermetically sealed containers for one week until further tests.

### 2.3. Evaluation of Phenolic Compound Composition (UPLC-ESI-MS/MS Conditions)

The variability in the qualitative and quantitative content of phenolic compounds in Japanese quince leaf samples was evaluated by applying validated UPLC-ESI-MS/MS method [17]. Samples were analyzed with Acquity H-class UPLC system (Waters Corporation, Milford, MA, USA) coupled with triple quadrupole tandem mass spectrometer (Xevo, Waters Corporation, Milford, MA, USA). To obtain MS/MS data an electrospray ionization source (ESI) was used. Compounds of interest were separated with YMC Triart C18 (100 × 2.0 mm; 1.9 μm) column (YMC Europe Gmbh, Dislanken, Germany). Constant temperature of 40 °C and flow rate of 0.5 mL·min^−1^ were maintained during analysis. Mobile phase consisted of 0.1% formic acid solution in water (solvent A) and acetonitrile (solvent B). Gradient profile was applied with following proportions of solvent A: Initially 95% for 1 min followed by linear increase to 70% over 4 min; 50% over next 3 min and to 95% over last 2 min. Analysis was performed in negative electrospray ionization mode. Capillary voltage was set to negative 2 kV. Temperature in ion source was maintained at 150 °C. Nitrogen gas temperature was set to 400 °C and flow rate to 700 L·h^−1^. Cone gas flow rate was set to 20 L·h^−1^. Each compound of interest had a specific collision energy and cone voltage selected. The selected mass spectrometry parameters for this method are presented in Table 1. The validation characteristics of the developed method are presented as supplementary Table S1.

### 2.4. C6 and HROG36 Cell Culture

The C6 and HROG36 cell lines were purchased from the Cell Lines Service GmbH (Germany). The cells suspended in DMEM with 10% of foetal bovine serum, 100 U/mL penicillin and streptomycin, seeded in 75 cm^2^ flasks, and incubated at 37 °C, with 5% CO_2_ and saturated humidity. The cells were reseeded to new flasks every 3 days. Twenty-four hours prior to the treatment with quince leaf extracts, the cells were transferred to 96 well plates (VWR) at density of 0.2 × 10^6^ cells/cm^2^.

### 2.5. Primary Neuronal-Glial Cell Culture

Primary rat cerebellar-glial cell culture was prepared as described previously [18]. Briefly, the cerebella were isolated, minced, and triturated in Versene solution (1:5000) to a single-cell suspension. The suspension was centrifuged at 270× *g* for 5 min and resuspended in DMEM with Glutamax supplemented with 5% horse serum, 5% foetal calf serum, 38 mM glucose, 25 mM KCl, and antibiotic-antimycotic solution. The cells were plated at a density of 0.25 × 10^6^ cells/cm^2^ in 96-well plates (VWR) coated with 0.0001% poly-L-lysine and kept in a humidified incubator containing 5% CO_2_ at 37 °C. The cultures were subjected to treatment after 7 days in vitro.

### 2.6. Treatments of the Cells with Quince Leaf Extracts

HROG36, C6, and primary cerebellar neuronal glial cells were treated with 0.88, 1.25, 1.63, 2.00, 2.38, 2.75, 3.13 and 3.75 mg/mL ethanolic extracts made from leaves of Japanese quince cultivars ‘Rasa’, ‘Darius’ or ‘Rondo’ for 24 h. The controls with the same volume of the solvent (ethanol) were made in parallel. In addition, the C6 and HROG36 cells were treated with temozolomide concentration range from 0.02 to 4.85 mg/mL, chlorogenic acid (range 5–500 g/mL), epicatechin (5–300 g/mL), hyperoside (5–333 g/mL), and quercitrin (2–220 g/mL). After treatment, the cells were subjected to viability evaluation.

### 2.7. Evaluation of Cellular Viability

Viability of C6, HROG36, and primary cerebellar neuronal glial cells after treatments was evaluated according to metabolic activity by means of PrestoBlue™ Cell Viability Reagent (Thermo Fisher Scientific). The fluorescence of resorufin produced after PrestoBlue reagent cleavage was measured in a plate reader Infinite M Plex (Tecan Austria, Salzburg, Austria) at excitation and emission wavelengths of 560 and 590 nm, respectively. The results were expressed as percentage of the untreated control fluorescence level.

In addition, C6 and primary cerebellar neuronal-glial cells were evaluated for necrosis by double-staining with Hoechst 33,342 (15 µg/mL, Merck) and propidium iodide (PI; 5 µg/mL, Merck). After 15 min incubation with the dyes in dark at room temperature, the nuclear fluorescence was assessed under fluorescent microscope OLYMPUS IX71SIF-3 (Olympus Corporation, Tokyo, Japan). Hoechst33342-only-positive nuclei exhibiting blue fluorescence were considered viable, and Hoechst3334-plus-PI-positive nuclei stained magenta (because of blue and red signal overlay) were identified as necrotic.

### 2.8. Statistical Analysis

The phenolic compound content of each cultivar was expressed as means ± SD (standard deviation) of three replicates. The significant differences (*p* ≤ 0.05) between means were evaluated using Tukey’s HSD (Honest Significant Difference test). Cellular viability and metabolic activity results are presented as means ± standard deviation of 5 experimental repeats, each of 3 technical repeats. The data are expressed as percentage of the untreated control. Statistical analysis was performed by one-way analysis of variance (ANOVA) with the Dunnett’s post-test by SigmaPlot 13.0 software (Systat Software Inc., Surrey, UK). A value of *p* < 0.05 was taken as the level of significance. EC_50_ was calculated by SigmaPlot 13.0 (Systat Software Inc., Surrey, UK) software by means of four-parameter logistic function. Correlations were analyzed by Microsoft Office Excel 2010 (Microsoft, Redmond, WA, USA) software Correlation function.

## 3. Results

### 3.1. Phenolic Compound Composition of Japanese Quince Leaves

The sum of detected phenols varied significantly between cultivars, the highest amount was found in ‘Darius’, and the lowest in ‘Rondo’ leaves (Table 2). Sixteen phenolic compounds were identified in the leaves of ‘Rondo’, while 15 in ‘Darius’, and 14 in ‘Rasa’. The majority of the identified phenols belong to three groups: Hydroxycinnamic acid derivatives, flavonols, and flavan-3-ols. There were also others, such as flavone luteolin 7-O-glucoside, and dihydrochalcone phloridzin. Total amount of hydroxycinnamic acid derivatives ranged from 5533 (‘Darius’) to 5839 (‘Rasa’) µg/g, and consisted of chlorogenic acid, p-coumaric acid, and caffeic acid. The flavan-3-ol group members were (-)-epicatechin, procyanidin B2, procyanidin C1, and (+)-catechin, and their total amount ranged from 700.4 (‘Rondo’) to 6426 (‘Darius’) µg/g. The flavonols found in the extracts were rutin, isoquercitrin, avicularin, kaempferol 3-O-glucoside, hyperoside, and quercetin. The total amount of the flavonols ranged from 2506 (‘Rondo’) to 4872 (‘Darius’) µg/g. The predominant group of phenols in ‘Rondo’ and ‘Rasa’ was the hydroxycinnamic acids, but in ‘Darius’ was flavan-3-ols. However, the total amount of hydroxycinnamic acids did not differ between the cultivars.

The total flavan-3-ol levels varied significantly between the cultivars. The highest amount was detected in ‘Darius’ leaves (6426 ± 145 µg/g), while ‘Rasa’ and ‘Rondo’ had around two and nine-fold less of the flavan-3-ols (3652 ± 73.6 µg/g and 700.4 ± 10.7 µg/g, respectively).

The total amount of flavonols was also significantly different between the cultivars. In addition, the distribution of individual compounds of this phenol group was different, too. The leaves of ‘Darius’ had significantly more isoquercitrin and quercitrin, ‘Rondo’ had more kaempferol 3-O-glucoside and luteolin 7-O-glucoside, and ‘Rasa’ had higher rutin amount. The main phenolic glycosides found in the extracts were isoquercitrin, hyperoside, and quercitrin.

### 3.2. The Effect of Quince Leaf Extracts on Viability of C6 and HROG36 Glioblastoma Cells

**Figure 1:**
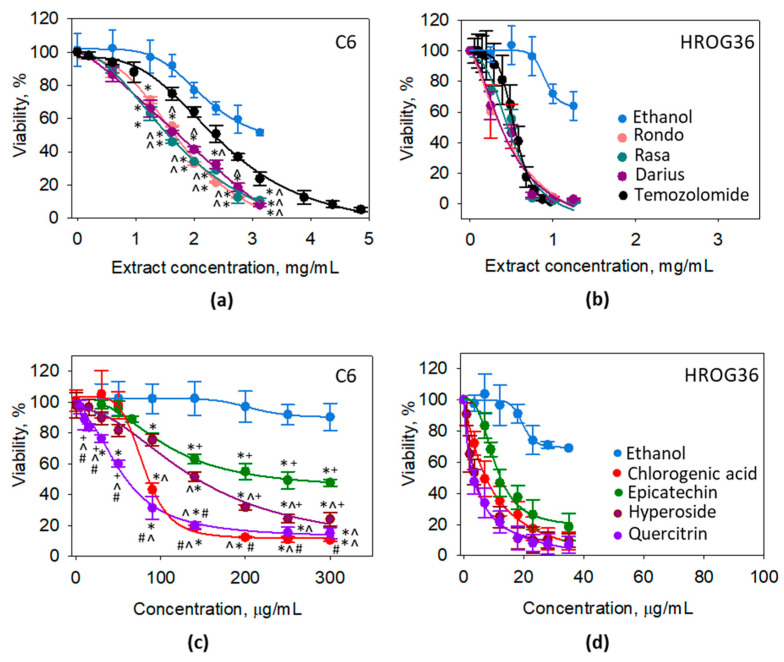
The effect of the extracts from leaves of different Japanese quince cultivars (**a**,**b**) and some phenolic compounds found in the extracts (**c**,**d**) on viability of glioblastoma C6 (**a**,**c**) and HROG36 (**b**,**d**) cells evaluated by metabolic activity assay with PrestoBlue reagent. For Ethanol, the concentrations were the same as in other samples at the indicated concentration point, i.e., 35, 50, 65, 80, 95, 110, 125, and 150 L/mL. In both (**a**,**c**), * indicates significant difference compared with ethanol-only treated samples; in (**a**), ^ significant difference compared with temozolomide; in (**c**), ^ significant difference compared with Epicatechin, + with Chlorogenic acid, # with Hyperoside. In (**b**), all extract-treated samples were statistically significantly different from Ethanol starting from concentration 0.25 mg/mL, and Temozolomide–starting from 0.5 mg/mL. in (**d**), all the samples were statistically significantly different from Ethanol starting from the concentration of 7 g/mL, and there was statistically significant difference between Epicatechin and other samples at 7 and 9 g/mL. The level of significance *p* < 0.05.

Next in the study, we evaluated the effect of the extracts from leaves of Japanese quince cultivars ‘Darius’, ‘Rasa’, and ‘Rondo’ on metabolic activity of rat C6 and human HROG36 glioblastoma cells (Figure 1).

After 24-h application of 1.25 mg/mL quince leaf extracts, the viability of C6 cells (assessed as cellular metabolic activity by PrestoBlue assay) was significantly decreased compared to ethanol control. The viability was by 14%, 11%, and 13% lower after treatment with ‘Rondo’, ‘Rasa’, and ‘Darius’, respectively (Figure 1a). The metabolic activity of the cells continued to decrease with increase in the concentration of the extracts and reached 8% of control in 3.125 mg/mL ‘Rondo’- and ‘Darius’-treated samples, and 10% in 3.125 mg/mL ‘Rasa”-treated samples. The difference between ethanol control and the 3.125 mg/mL extract-treated samples were 43%, 41%, and 43% for ‘Rondo’, ‘Rasa’, and ‘Darius’, respectively. The metabolic activity of C6 cells was more sensitive to the extracts compared to the effect of temozolomide, the drug used for glioblastoma treatment. The metabolic activity of C6 cells treated with extracts at 1.25 mg/mL and higher concentration was significantly lower than in the C6 samples treated by the same concentrations of temozolomide. This was also reflected in calculated EC_50_; the values of ‘Rondo’, ‘Rasa’, and ‘Darius’ were 71%, 67%, and 74% lower compared to the EC_50_ value of temozolomide for C6 cells (Table 3).

Human glioblastoma cells HROG36 were more sensitive to quince leaf extract treatment compared to C6 cells (Figure 1b). ‘Rondo’, ‘Rasa’, and ‘Darius’ applied at 0.25 mg/mL significantly decreased metabolic activity of HROG36 cells compared to ethanol control by 37%, 24%, and 34%, respectively. After treatment with 0.75 mg/mL extracts, metabolic activity of HROG36 cells was less than 5% and about 91% lower compared to the ethanol control. The effect of temozolomide on HROG36 cell metabolic activity was similar to that of the extracts, and there were no statistically significant differences detected. However, the EC_50_ value of temozolomide calculated from the average titration data was by 157.3–162.0 μg/mL higher compared to the EC_50_ of the extracts (Table 3).

For the next step in the study, we have investigated the toxicity of the phenolic compounds identified in the quince leaf extracts on C6 and HROG36 cells. Chlorogenic acid was selected as a representative of hydroxycinnamic acids, hyperoside and quercitrin from flavonols, and epicatechin from flavan-3-ols. C6 cells were most sensitive to quercitrin and chlorogenic acid, as presented in the titration curves in Figure 1c and EC_50_ values in Table 3. The next least toxic compound was hyperoside, and the least toxic was epicatechin. Similarly to the case of extract treatment, HROG36 cells were more sensitive to the phenolic compounds compared to the C6 cells (Figure 1c,d). After treatment with 23 μg/mL of each compound, the metabolic activity of HROG36 cells was 8% (for quercitrin)–26% (for epicatechin) of the untreated control. For comparison, after similar 30 μg/mL treatment in C6 cell samples, the metabolic activity was either unchanged (chlorogenic acid, epicatechin), or decreased only to 90% (hyperoside) and 75% (quercitrin) of untreated control. The most toxic for HROG36 cells were quercitrin and hyperoside, although chlorogenic acid was also very close to that level. A slightly lower toxicity was caused by epicatechin; there was statistically significant difference between epicatechin and other investigated phenolic compounds at 7 and 9 g/mL.

The potential input of each group of phenolic compounds and some individual phenols in the extracts was estimated by correlation analysis (Table 4).

Strong negative correlation with r value close to −1 was found between viability level of both glioblastoma cell types and amounts of hydroxycinnamic acids and chlorogenic acid. In addition, strong negative correlation was between metabolic activity of C6 and hyperoside, and between metabolic activity of HROG36 and quercitrin. Moderate negative correlation was between C6 viability and the amount of quercitrin, and low negative—between HROG36 viability and total contents of flavonols and phenols. The analysis allows to suggest that the toxicity of the extracts was most likely mediated by chlorogenic acid.

**Figure 2:**
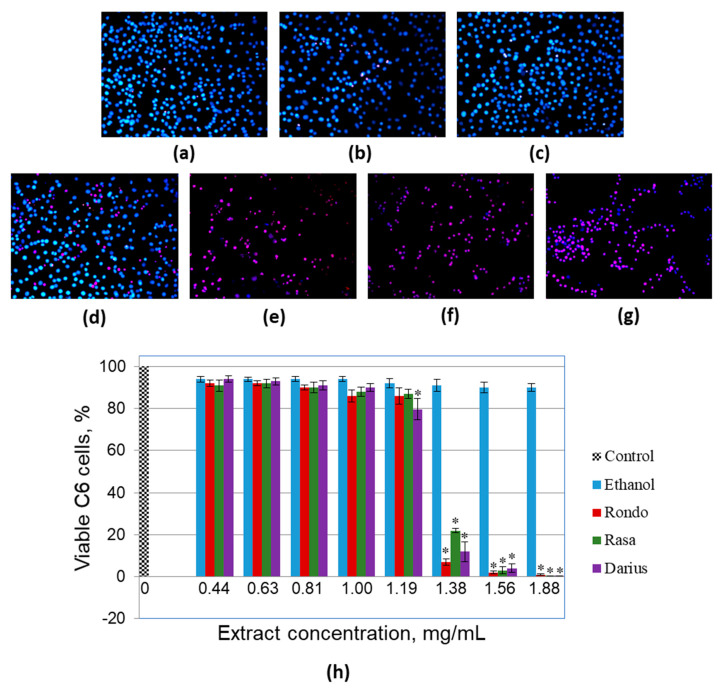
The effect of the extracts from leaves of different Japanese quince cultivars on viability of glioblastoma C6 cells. (**a**–**g**) Characteristic images of Hoechst/propidium iodide staining of the C6 cell nuclei after extract treatments; (**a**) samples treated with 125 µL/mL of the solvent ethanol (amount corresponds to 1.88 mg/mL extract treatment); (**b**–**d**) treated with 1.19 mg/mL extracts from ‘Rondo’, ‘Rasa’, and ‘Darius’ cultivars, respectively; (**e**–**g**) samples, after treatment with 1.56 mg/mL extracts from ‘Rondo’, ‘Rasa’, and ‘Darius’ cultivars, respectively; (**h**) quantitative summary of viability data presented as averages with standard deviation, * indicates significant difference compared with ethanol-only treated samples, *p* < 0.05.

Metabolic activity assays such as PrestoBlue reflects not only changes in cell viability, but also differences in proliferation rate and metabolic disturbances. Therefore, we have additionally investigated viability of C6 cells after quince leaf extract treatments by double nuclear fluorescence staining that allows to detect necrotic cells with lost membrane integrity (Figure 2).

The extracts did not significantly affect C6 cell viability up to the concentration of 1.19 mg/mL (Figure 2b–d,h). Treatment with 1.19 mg/mL extract from leaves of ‘Darius’ induced small yet significant decrease in percentage of viable C6 cells (Figure 2d,h). The average level of viable cells in ‘Darius’ extract-treated samples was by 14% lower compared with samples treated with the same amount of ethanol. Further increase in extract concentration to 1.38 mg/mL caused a remarkable drop in C6 viability in all three cultivar groups (Figure 2h). The average level of viable cells decreased by 93%, 78%, and 88% after treatment with extracts from ‘Rondo’, ‘Rasa’, and ‘Darius’, respectively. After treatment with 1.88 mg/mL extracts, the number of viable cells in C6 samples was close to ‘zero’ in all three cultivar groups. There was no significant decrease in C6 cell viability, observed after treatment with ethanol up to 1.88 mg/mL. Calculated levels of EC_50_ from double nuclear staining experiments were 1.26 mg/mL, 13.0 mg/mL, and 1.26 mg/mL for ‘Rondo’, ‘Rasa’, and ‘Darius’, respectively; the values slightly lower yet similar to those revealed by metabolic activity assay.

### 3.3. The Effect of Quince Leaf Extracts on Viability of Primary Non-Cancerous Brain Cells

**Figure 3:**
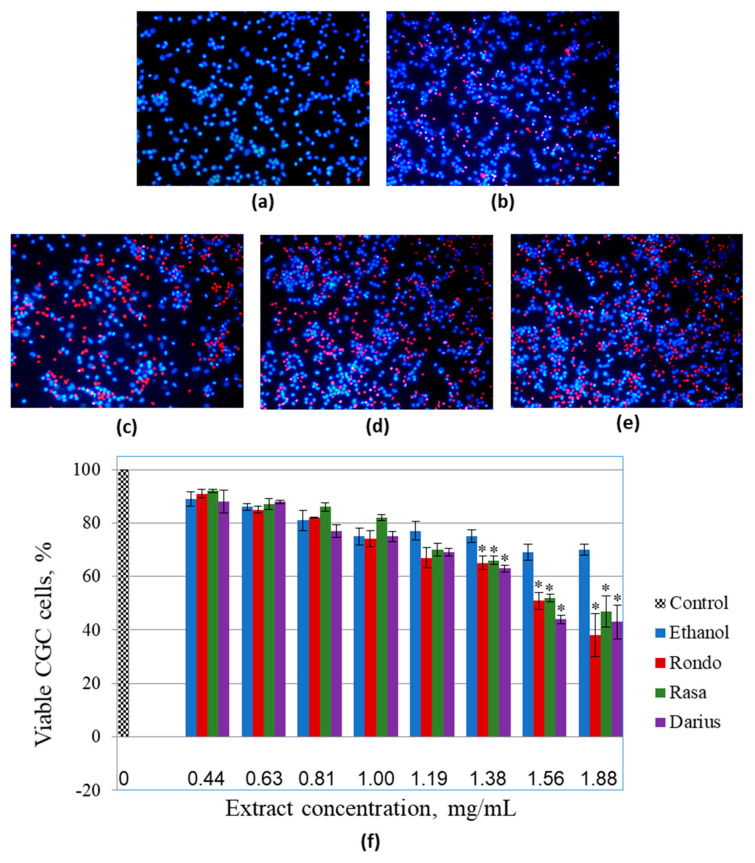
The effect of the extracts from leaves of different Japanese quince cultivars on viability of cultivated primary rat cerebellar neuronal-glial cells. (**a**–**e**) Representative images of nuclei stained with Hoechst33342 and propidium iodide after extract treatments: (**a**) Untreated control, (**b**) sample treated with 125 µL/mL of the solvent ethanol; (**c**–**e**) samples treated with 1.19 mg/mL extracts from ‘Rondo’, ‘Rasa’, and ‘Darius’ cultivars, respectively; (**f**) quantitative summary of viability data presented as averages with standard deviation, * indicates significant difference compared with the samples treated with the same amount of ethanol.

All the Japanese quince leaf extracts investigated in the study were toxic to human rat glioblastoma C6 cells at concentrations equal to or higher than 1.38 mg/mL. Human glioblastoma HROG36 cells were even more sensitive to the treatments. The next step in this study was to investigate whether primary non-cancerous brain cells have similar susceptibility to the same extract treatment (Figure 3).

For these experiments, rat cerebellar neuronal-glial cell cultures consisting of approximately of 81 ± 4% granule neurons, 14 ± 3% astrocytes, and 6 ± 2% microglial cells [18] were used. The extracts up to the concentration of 1.0 mg/mL did not cause significant decrease in viability of primary cerebellar cells compared to the respective ethanol control (Figure 3f). However, increase in concentration up to 1.19 mg/mL caused viability drop by 13% (‘Rondo’), 9% (‘Rasa’), and 10% (‘Darius’) compared with the respective ethanol treatment. Further increase in extract concentration continued to lower the number of viable cells, and after treatment with 1.88 mg/mL extracts, the average numbers such cells in primary neuronal-glial cultures were 38%, 47%, and 43% for ‘Rondo’, ‘Rasa’, and ‘Darius’, respectively. In the case of C6 glioblastoma cells, the extracts applied at concentration 1.56 mg/mL induced 80% and higher loss of viable cells, and there were almost no viable cells left after treatment with 1.88 mg/mL extracts (Figure 2). In primary brain cell cultures, the level of viable cells remained higher than 50% after treatment with 1.56 mg/mL extracts and close to 50% after treatment with 1.88 mg/mL extracts. Thus, primary neuronal-glial cells from rat cerebella were less sensitive to the toxic effect of the 1.56–1.88 mg/mL extracts from quince leaves compared to the glioblastoma C6 cells. This was also confirmed by the calculated EC_50_ of the extracts for primary cerebellar cells; the values were 1.58 mg/mL, 1.72 mg/mL, and 1.64 mg/mL for ‘Rondo’, ‘Rasa’, and ‘Darius’, respectively, and they were higher than the values for C6 cells calculated from the double nuclear staining assay data. However, primary neuronal-glial cells were more sensitive to the solvent ethanol compared to C6 cell line. There was significant decrease in primary cerebellar cell viability observed after treatment with 80 µL/mL ethanol; the average level of viable cells after this treatment was by 25% lower compared to the untreated control. After treatment with 150 µL/mL ethanol, the percentage of viable cells were by 30% lower than in control samples.

## 4. Discussion

Japanese quince leaf extract consisted of three major phenol groups: Hydroxycinnamic acids, flavan-3-ols, and flavonols. However, distribution of these groups between cultivars was significantly different. For example, in ‘Rondo’, more than half of total phenols consisted of hydroxycinnamic acid derivatives (62.6%), while ‘Darius’ and ‘Rasa’ contained only 32.5%, and 43% of these compounds, respectively. Similar amounts (from 42.90% to 50.90%) of hydroxycinnamic acids in quince leaves were found in a recent study of Chojnacka and co-authors [13]. The predominant compound of this group in all cultivars was chlorogenic acid, and this is in agreement with other studies [13,19]. The amount of flavonols in all cultivars was around 30% of total phenols. The majority of the flavonols consisted of isoquercitrin, hyperoside, and quercitrin, all the three are known for anti-cancer properties [20,21,22]. Saccharide moiety of the compounds mediates the toxicity interacting with membranes of cancer cells and promoting active uptake of the compounds [23,24]. The distribution of flavan-3-ols between cultivars was most diverse and varied from 7.5% (‘Rondo’) to 37.7% (‘Darius’).

In the present study, we have found that ethanolic quince leave extracts were cytotoxic similarly as (in the case of human glioblastoma HROG36 cells) or even more than (in rat C6 cell case) temozolomide, the drug used for glioblastoma treatment in the clinical practice. The viability of both investigated cell types had strong negative correlation with the amount of hydroxycinnamic acid derivatives and chlorogenic acid, and the cytotoxicity of the chlorogenic acid was also demonstrated in both C6 and HROG36 cell cultures. Although other investigated compounds were also toxic to the cells, especially flavonols quercitrin and hyperoside, the amounts of the compounds present in the extracts were far too small to mediate the toxicity for C6 cells. However, for HROG36 cells, the flavonols could contribute to the toxicity of chlorogenic acid in the extracts because the EC_50_ values of the compounds for the cells were close to the levels found in the extracts. The anticancer activity of hydroxycinnamic acids is also reported by others. These compounds promote apoptosis, arrest cell cycle, and prevent metastasis of different types of breast, lung, colon, gastric, liver, pancreatic, and prostate cancer cells [25,26,27]. Ekbatan and colleagues have shown that chlorogenic acid and its metabolites caffeic acid, 3-phenyl propionic acid, and benzoic acid cause cell cycle arrest and apoptosis of colon cancer cells Caco 2 [28]. Another group of scientists has demonstrated that chlorogenic acid disrupts cytoskeleton organization and mTORC2 signalling of both adenocarcinomic human alveolar basal epithelial cells A549 and human hepatocyte carcinoma HepG2 [29]. The above-mentioned findings are in line with another extensive study performed by Huang and co-workers, who investigated anticancer activity of chlorogenic acid on human cancerous lung, liver, kidney, colon, and brain cells including human glioblastoma lines U87MG and M059J, rat C6, and mouse G422 [30]. The study revealed that chlorogenic acid promotes all cancer cell (including glioblastoma) cycle arrest and differentiation to maturation phenotype via miR-17 family downregulation, p21 upregulation and mitochondrial suppression. The efficiency of chlorogenic acid was comparable to that of temozolomide.

Comparison of viability of primary rat non-cancerous brain cells and rat glioblastoma C6 cells evaluated by double nuclear staining revealed the significantly higher sensitivity of glioblastoma for the quince leave extracts. Although the EC_50_ values of the extracts for the non-cancerous cells were similar to those calculated for C6 cells from the metabolic PrestoBlue evaluation data, to our opinion, it would not be relevant to compare the data obtained from different viability assessment assays. PrestoBlue assay monitors the rate of cellular metabolic activity, which is proportional to the number of viable cells. However, the metabolic activity might be directly influenced by the investigated compound without causing cell death, e.g., chlorogenic acid, the main component of the quince leaf extract, is reported to decrease mitochondrial activity of glioblastoma [30]. The number of viable cells might be lower not, or not only, due to the increase in cell death, but also due to the suppression of proliferation, because hydroxycinnamic acids present in the extracts might induce cell cycle arrest [26]. Thus, the metabolic assay is different from the evaluation of viability by double nuclear staining which gives information about percentage of necrotic and viable cells without sensing the metabolic activity or proliferation rate of them.

Similar finding about lower susceptibility to quince leaf extract of non-cancerous cells compared to cancer cells was recently described by Chojnacka and co-authors [13]. Such higher sensitivity of cancer cells to bioactive compounds of quince leaf extracts could be related to specific biology of these cells. Usually, cancer cells have higher proliferation rate, migration capacity and altered energy metabolism compared to the surrounding non-cancerous cells [31,32]. Such a difference opens a niche for selective targeting of cancer cells with lower risk to destroy healthy non-cancerous cells. Ethanolic extracts from Japanese quince leaves have several compounds that interfere with cancer cell-specific pathways related to proliferation, migration and energy metabolism [4,29,30]. This might at least partially explain cancer cell-selective cytotoxicity of quince leaf extracts.

Thinking about quince leaf extracts as complementary therapy for glioblastoma, it is important to evaluate the ability of the extract compounds to cross the blood-brain barrier (BBB). One of the best studied pathways for plant phenolic metabolites to cross BBB is passive permeation, however active transport might be also possible. Lee and co-authors have found that intraperitoneally administered chlorogenic acid ameliorates brain damage and oedema after cerebral ischaemia in rats [33]. In another study, chlorogenic acid was found both in the blood and brain after intraperitoneal administration in mice, and was safe even at very high doses (1000 mg/kg) [30]. A pharmacokinetics and brain penetration study shows that chlorogenic acid is rapidly absorbed in plasma after both intranasal and intravenous administration of 10 mg/kg and reaches brain and cerebrospinal fluid [34]. The concentration of chlorogenic acid in the brain after 30 min of intranasal administration was about 250 g/mL and remained about 25 g/mL after 6 h. Such data allow to suggest that chlorogenic acid and other compounds from quince leave extracts might be applied as complementary therapy for glioblastoma. However, future research should focus on an effective and safe dose, biologically active compound absorption, distribution, and excretion.

## 5. Conclusions

The main compound in ethanolic extracts from Japanese quince cultivars ‘Rondo’, ‘Rasa’, and ‘Darius’ is chlorogenic acid, and the amount of this compound is similar in all three cultivars. The amount of other phenolic compounds is more variable between the cultivars. The extracts of the leaves of all three cultivars significantly decrease viability of C6 and HROG36 glioblastoma cells; in the case of the HROG36 cells, the extracts are equally toxic, but in the C6 cells, the extracts are more toxic than glioblastoma drug temozolomide. The effect on viability has strong correlation with the level of chlorogenic acid for both cell types. In addition, quince leaf extracts exert significantly higher toxicity on rat C6 glioblastoma cells compared to primary rat neuronal-glial cerebellar cells. This finding suggests that Japanese quince leaves could be further investigated as anticancer drugs for glioblastoma treatment.

## Figures and Tables

**Table 1:** Mass spectrometry parameters for the analysis of phenolic compounds.

Compound	Retention Time, min	Molecular Ion (*m*/*z*)	Production (*m*/*z*)	Cone Voltage, V	Collision Chamber Energy, eV
(+)-Catechin	3.50	289	123	60	34
Chlorogenic acid	3.52	353	191	32	14
Caffeic acid	3.89	179	107	36	22
Procyanidin B2	4.02	577	407	50	20
(-)-Epicatechin	4.13	289	123	60	34
Procyanidin C1	4.38	865	125	56	60
p-Coumaric acid	4.73	163	93	28	22
Rutin	5.06	609	300	70	38
Hyperoside	5.20	463	300	50	26
Isoquercitrin	5.30	463	301	52	28
Luteolin 7-O-glucoside	5.31	447	285	66	26
Avicularin	5.59	433	301	50	20
Kaempferol 3-O-glucoside	5.64	447	284	54	28
Quercitrin	5.68	447	300	50	26
Phloridzin	5.88	435	273	42	14
Quercetin	6.86	301	151	48	20

**Table 2:** Quantitative composition of phenols in Japanese quince leaves, µg/g DW.

Compound, µg/g DW	Japanese Quince Cultivars		
	‘Darius’	‘Rondo’	‘Rasa’
Hydroxycinnamic Acids			
Chlorogenic acid	5373 ± 244 ^a^	5773 ± 271 ^a^	5737 ± 269 ^a^
p-Coumaric acid	155.4 ± 10.2 ^a^	58.9 ± 3.3 ^c^	102.9 ± 7.5 ^b^
Caffeic acid	4.8 ± 0.2 ^a^	0.84 ± 0.03 ^b^	ND
Total, µg·g^−1^	5533 ± 202 ^a^	5833 ± 307 ^a^	5840 ± 188 ^a^
Flavonols			
Isoquercitrin	2131.1 ± 54.4 ^a^	418.6 ± 11.5 ^c^	1700.7 ± 49.6 ^b^
Hyperoside	907.7 ± 40.3 ^b^	1124.5 ± 51.4 ^a^	1107.8 ± 50.3 ^a^
Quercitrin	1314.3 ± 39.2 ^a^	248.5 ± 10.1 ^c^	350.0 ± 20.2 ^b^
Rutin	189.7 ± 9.0 ^c^	289.1 ± 13.2 ^b^	511.2 ± 20.0 ^a^
Kaempherol 3-O-glucoside	318.0 ± 14.3 ^b^	407.9 ± 18.3 ^a^	226.9 ± 10.3 ^c^
Avicularin	9.34 ± 0.32 ^b^	14.97 ± 0.63 ^a^	5.69 ± 0.20 ^b^
Quercetin	2.14 ± 0.09 ^a^	2.42 ± 0.10 ^a^	1.23 ± 0.04 ^b^
Total, µg·g^−1^	4872 ± 140 ^a^	2506 ± 56 ^c^	3903 ± 14.3 ^b^
Flavan-3-Ols			
(-)-Epicatechin	3963 ± 112 ^a^	314.3 ± 9.4 ^c^	2038 ± 88 ^b^
Procyanidin C1	1333.8 ± 53.5 ^a^	176.1 ± 8.4 ^c^	792.4 ± 21.1 ^b^
Procyanidin B2	1129.3 ± 50.3 ^a^	209.4 ± 15.6 ^c^	820.9 ± 33.6 ^b^
(+)-Catechin	ND	0.6 ± 0.02	ND
Total, µg·g^−1^	6426 ± 145 ^a^	700.4 ± 10.7 ^c^	3652 ± 73.6 ^b^
Others Phenols			
Luteolin 7-O-glucoside	213.7 ± 8.6 ^b^	277.9 ± 11.3 ^a^	155.5 ± 6.9 ^c^
Phloridzin	3.65 ± 0.16 ^b^	5.32 ± 0.23 ^a^	5.75 ± 0.25 ^a^
Sum of all compounds, µg/g	17048 ± 461 ^a^	9322 ± 287 ^c^	13556 ± 375 ^b^

Value is average ± SD (*n* = 3); Different letters in the same line indicate a statistically significant difference (*p* ≤ 0.05); DW: dry weight; ND: not detected.

**Table 3:** Calculated EC_50_ of extracts from leaves of quince cultivars ‘Rondo’, ‘Rasa’, and ‘Darius’ and of some phenolic compounds found in the extracts for C6 and HROG36 cells.

Substance, μg/mL	‘Rondo’	‘Rasa’	‘Darius’	Chlorogenic Acid	Epicate Chin	Hypero Side	Querci Trin	Temozo Lomide
C6	1660.2	1560.0	1738.9	85.5	252.2	143.1	59.3	2341.5
HROG36	373.8	378.5	373.5	7.3	12.3	3.8	3.4	535.8

**Table 4:** The values of coefficient for correlation between viability (metabolic activity) of C6 or HROG36 cells and amount of phenolic compounds in the extracts from leaves of quince cultivars ‘Rondo’, ‘Rasa’, and ‘Darius’.

Compound	Hydroxycinnamic Acids	Flavonols	Flavan-3-Ols	Total Phenols	Chlorogenic Acid	Epicate Chin	Hypero Side	Querci Trin
C6	−0.99	0.46	0.21	0.39	−0.99	0.33	−0.75	−0.56
HROG36	−0.86	−0.38	0.28	−0.42	−0.81	−0.21	−0.31	−0.81

## Data Availability

The data presented in this study are available on request from the corresponding author. The data are not publicly available due to the institutional data policy.

## Supplementary Materials

The following are available online at https://www.mdpi.com/2304-8158/10/1/18/s1, Table S1: Validation characteristics of developed UPLC-ESI-MS/MS method.
