# Supplementary material for: Investigation of Phenolic Composition and Anticancer Properties of Ethanolic Extracts of Japanese Quince Leaves
Source: Foods. 2020 Dec 23;10(1):18. doi: 10.3390/foods10010018 (PMC7822480; doi:10.3390/foods10010018)
Supplement: Supplementary file 1 [file foods-10-00018-s001.pdf]

Table S1. Validation characteristics of developed UPLC-ESI-MS/MS method

Compound	Accuracy (n=6), RSD %	Inter-day Precision (n=6), RSD %	Limit of detection, ng/mL	Limit of quantification, ng/mL	Range of linearity, µg/mL	Calibration curve	R^2
**(+)-Catechin**	5.31	7.68	24.08	72.26	0.25 - 500	Y=78.29*X + 6.69	0.97
**Chlorogenic acid**	5.34	7.65	1.05	3.55	0.313 – 625	Y=1796.14*X + 543.97	0.99
**Caffeic acid**	6.82	5.25	2.91	11.54	0.313 – 625	Y=3824.7*X + 1524.63	0.96
**Procyanidin B2**	3.71	5.67	12.06	36.18	0.313 – 625	Y=260.327*X+72.139	0.98
**(-)-Epicatechin**	5.84	6.31	32.51	97.55	0.25 - 500	Y=137.66*X + 40.66	0.98
**Procyanidin C1**	6.23	9.17	6.5	20	0.313 – 625	Y=174.108*X+42.742	0.99
**p-coumaric acid**	3.92	2.7	4.6	15.35	0.313 – 625	Y=2851.9*X + 805.23	0.98
**Quercetin-3-O-rutinoside (Rutin)**	3.16	4.21	2.2	6.8	0.313 – 625	Y=1690.74*X + 349.09	0.99
**Hyperoside**	6.62	9.28	1.5	5	0.313 – 625	Y=5041.08*X+1328.9	0.96
**Quercetin 3-O-glucoside (Isoquercitrin)**	3.19	5.63	1.2	4.52	0.313 – 625	Y=2301.1*X – 456.94	0.97
**Luteolin-7-O-glucoside (Cynaroside)**	4.23	3.86	0.8	2.8	0.25 - 500	Y=9124.67*X + 1082.01	0.99
**Quercetin 3-O-α-L-arabinofuranoside (Avicularin)**	5.38	8.35	0.83	2.85	0.313 – 625	Y=2683.43*X + 1168.7	0.97
**Kaempherol-3-O-glucoside (Astragalin)**	2.95	3.63	1.05	3.5	0.25 - 500	Y=8805.36*X + 988.2	0.99
**Quercetin 3-O-rhamnoside (Quercitrin)**	3.42	5.32	0.99	3.2	0.313 – 625	Y=2200.8*X- 803.678	0.98
**Phloretin-2'-O-glucoside (Phloridzin)**	3.52	5.32	0.6	1.94	0.313 – 625	Y=2388.71*X + 2183.37	0.99
**Quercetin**	4.73	5.91	3.5	10.51	0.25 - 500	Y=2638.29*X- 299.64	0.99
